# Peroxiredoxin 3 Inhibits Acetaminophen-Induced Liver Pyroptosis Through the Regulation of Mitochondrial ROS

**DOI:** 10.3389/fimmu.2021.652782

**Published:** 2021-05-13

**Authors:** Yue Wang, Yan Zhao, Zhecheng Wang, Ruimin Sun, Boyang Zou, Ruixi Li, Deshun Liu, Musen Lin, Junjun Zhou, Shili Ning, Xiaofeng Tian, Jihong Yao

**Affiliations:** ^1^ Department of Pharmacology, Dalian Medical University, Dalian, China; ^2^ Institute of Integrative Medicine, Dalian Medical University, Dalian, China; ^3^ Department of General Surgery, The Second Affiliated Hospital of Dalian Medical University, Dalian, China; ^4^ Department of Pharmacy, The Second Affiliated Hospital of Dalian Medical University, Dalian, China

**Keywords:** PRX3, pyroptosis, mitochondrial ROS (mtROS), NLRP3 inflammasome, APAP

## Abstract

Pyroptosis is a newly discovered form of cell death. Peroxiredoxin 3 (PRX3) plays a crucial role in scavenging reactive oxygen species (ROS), but its hepatoprotective capacity in acetaminophen (APAP)-induced liver disease remains unclear. The aim of this study was to assess the role of PRX3 in the regulation of pyroptosis during APAP-mediated hepatotoxicity. We demonstrated that pyroptosis occurs in APAP-induced liver injury accompanied by intense oxidative stress and inflammation, and liver specific PRX3 silencing aggravated the initiation of pyroptosis and liver injury after APAP intervention. Notably, excessive mitochondrial ROS (mtROS) was observed to trigger pyroptosis by activating the NLRP3 inflammasome, which was ameliorated by Mito-TEMPO treatment, indicating that the anti-pyroptotic role of PRX3 relies on its powerful ability to regulate mtROS. Overall, PRX3 regulates NLRP3-dependent pyroptosis in APAP-induced liver injury by targeting mitochondrial oxidative stress.

## Introduction

Acetaminophen (APAP) is one of the most commonly used analgesic and antipyretic drugs. The recommended dose is safe and effective, but overdose causes hepatotoxicity and acute liver failure (ALF) in a dose-dependent manner (1). The initial step in APAP-mediated hepatotoxicity is the formation of the highly reactive intermediate metabolite n-acetyl-p-benzoquinone imine (NAPQI). Excessive formation of NAPQI consumes intracellular glutathione, binds to mitochondrial proteins, and impairs mitochondrial respiration, which subsequently gives rise to overwhelming mitochondrial oxidative stress that triggers signaling pathways through mitochondrial toxicity, eventually leading to cell death and sterile inflammation (2, 3). Importantly, the excessive activation of immune cells, such as Kupffer cells (KCs) causes an inability to shut down the persistent activation of the inflammatory response, which results in tissue damage and disease progression (4–7). Recent studies have shown that APAP displays dose-dependent toxicity in primary hepatocytes. Moreover, upon APAP overdose in mice, at least 50% of KCs exhibit a significant loss (8–10). However, the molecular mechanism of liver cell death in APAP-induced hepatotoxic processes needs to be further elucidated.

Pyroptosis is a newly identified type of programmed cell death (PCD) characterized by pore formation in the plasma membrane, swelling, cell rupture, and proinflammatory factor release (11, 12). As early as 1992, Zychlinsky et al. initially observed pyroptosis and described a lytic form of cell death in macrophages infected by *Shigella flexneri* (13). Pyroptosis is related to innate immune activation, participates in sterile inflammation, and contributes to the development of acute and chronic liver disease (14, 15). To date, the relevance of pyroptosis in APAP-mediated liver damage remains enigmatic. It has been suggested that the NLRP3 inflammasome activates pyroptosis in several diseases, including atherosclerosis, Alzheimer’s disease, acute kidney and liver diseases (16–21). Reactive oxygen species (ROS) from damaged mitochondria are the main activator of NLRP3, and the mitochondria serve as the docking site for inflammasome assembly (22–24). Nevertheless, there are no studies on the molecular mechanism linking APAP-induced mitochondrial oxidative stress and pyroptosis.

Peroxiredoxins (PRXs) are a family of thiol peroxidases that scavenge peroxides in cells. Mammals have six PRXs, of which PRX3 is specifically localized in the mitochondria that is the main site for the formation of peroxynitrite and hydrogen peroxide. As the most abundant peroxidase in most mouse tissues, PRX3 not only eliminates a large amount of hydrogen peroxide but also functions as the main target of peroxynitrite in the mitochondria (25, 26). Previous studies have shown that PRX3-knockout mice accumulate relatively high intracellular ROS levels, which increases the severity of LPS-induced lung injury (27). Moreover, PRX3 deficiency accelerates chronic kidney disease by promoting fibrosis and inflammation accompanied by mitochondrial oxidative stress (28). In contrast, PRX3 overexpression in mice protects against traumatic neuronal injury by preserving mitochondrial function and mitochondrial biogenesis (29) and prevents mitochondrial oxidative damage induced by intestinal ischemia/reperfusion (I/R) injury (30). However, the potential role of PRX3 in APAP-induced hepatotoxicity needs to be further clarified. Notably, a proteomic analysis of APAP-induced acute liver injury in mice showed that a decrease in PRX3 levels may be partly responsible for the excessive accumulation of ROS and aldehyde, which impairs mitochondrial structure and function (31). Therefore, determining the role of PRX3 may be helpful in the treatment of APAP-induced hepatotoxicity.

In this study, we hypothesized that pyroptosis participates in APAP-induced liver injury and elucidated the mechanism and function of PRX3 in this pathological process. These findings may provide new insights and a target for the treatment of APAP overdose.

## Materials and Methods

### Animal Experiments

Male C57BL/6 mice (10 weeks old, specific pathogen-free) were purchased from the Animal Center of Dalian Medical University (Dalian, China). Mice were housed in an environment maintained at 23 ± 2°C with ad libitum access to food and water under a 12-h light/dark cycle. APAP was purchased from Aladdin (Shanghai, China) and dissolved in warmed 37°C saline. To establish a murine model of APAP-induced ALF, totally 16 male C57BL/6 mice were randomly divided into 2 groups (n = 8) and received treatment as follows: a. control group: 24-h-fasted mice were treated with a control agent (normal saline). b. APAP group: 24-h-fasted mice were each given APAP (300 mg/kg) by oral gavage. The mice were sacrificed 12 h after normal saline or APAP gavage, and serum and liver tissues were collected and stored at -80°C for further analysis.

To generate a liver-specific knockdown of PRX3, PRX3-specific small short hairpin RNA (shRNA) was cloned and packaged into an adeno-associated virus 9 (AAV9) with the liver-specific thyroxin binding globulin (TBG) promoter (Hanbio, Shanghai, China). The AAV9-TBG-shRNA-NC/PRX3 was packaged with pAAV-RC and pHelper using the triple-plasmid transient transfection method (HB infusion^TM^ Kit; Hanbio Bio.). Viral particles were applied in the following animal experiments after purification. The shRNA-PRX3 sequence was as follows: 5’-AAGGTATATTGCTGTTGACAGTGAGCGCCAAGGAAAGTCAGCCTTTTAGTGAAGCCACAGATGTAAAAGGCTGACTTTCCTTGGTGCCTACTGCCTCG-3’. In the AAV9-shRNA-treated APAP liver injury model, mice were randomly divided into 4 groups (n = 8; totally 32 mice) and administered with 200 μl of AAV9-shRNA-NC/PRX3 (1.9*10^12^ vg/mL) through tail vein injection. At 3 weeks after injection, mice were used in experiments as mentioned before. After the administration of APAP for an additional 12 h, the livers and serum were harvested and analyzed.

All procedures were carried out in accordance with the Institutional Laboratory Animal Care and Use Guidelines and approved by the Institutional Ethics Committee of Dalian Medical University. Animal studies are reported in compliance with the ARRIVE guidelines (32).

### Measurement of Serum Parameters

Serum levels of alanine aminotransferase (ALT) and aspartic aminotransferase (AST) were measured with appropriate assay kits (Jiancheng Bioengineering Institute, Nanjing, China).

### Enzyme-Linked Immunosorbent Assay

Mice serum levels of IL-1β and IL-18 were assayed using an enzyme-linked immunosorbent assay (ELISA) kit (Jiancheng Bioengineering Institute, Nanjing, China) according to the manufacturer’s instructions.

### Lactate Dehydrogenase Assay

Serum and cellular supernatants were collected for a lactate dehydrogenase (LDH) assay (Jiancheng Bioengineering Institute, Nanjing, China) applied according to the manufacturer’s instructions. Optical density (OD) values were measured at 450 nm using a Thermo Multiskan FC microplate photometer.

### Histological Examination and Immunohistochemical Staining

Liver tissue sections were cut and fixed with 4% paraformaldehyde at least overnight, and then hematoxylin-eosin (H&E) staining was performed.

Immunohistochemistry (IHC) for PRX3 was performed using 4-μm-thick paraffin sections. Sections were incubated with an anti-PRX3 antibody (Abcam, Cambridge, UK) overnight at 4°C and then incubated with a secondary antibody (Servicebio, Wuhan, China) at room temperature for 50 min. Subsequently, the tissues were covered with DAB and hematoxylin, and sections were observed via light microscopy.

### Isolation and Culture of Primary Liver Cells

Primary KCs and hepatocytes were isolated from mice as described previously (33, 34). Briefly, after 12h fasting, the mice were anesthetized, sterilized with 75 % alcohol and fixed on an anatomical plate in the ultra-clean table. 25 mL CMF-HBSS was infused from the hepatic portal vein of mice, and then 25 mL Hank’s Balanced Salt Solution (HBSS) with 0.5 mg/ml collagenase IV (both from Solabio, Beijing, China) was infused until the liver failed to rebound after pressing. After digestion, the liver was dissected and placed in a petri dish with a cold HBSS buffer. The cell suspension was successively passed through 100- and 200-mesh filters, and then centrifuged three times at 50×g for 3 min to obtain hepatocytes. The initial cell supernatant was collected and centrifuged at 650×g at 4°C for 7 min, after which the pelleted cells were resuspended in 2ml HBSS. Then, the cell suspension was gently layered onto an 8ml two-step Percoll gradient (25%/50%; Solabio) in a 15 ml conical centrifuge tube, and the tube was centrifuged at 1800 g for 15 min at 4°C. The intermediate mesophase (white cell ring) was carefully collected, resuspended with HBSS and centrifuged at 650×g for 7 min to obtain the precipitated KCs. Isolated primary liver cells were seeded on rat tail collagen I-coated culture plates (Corning Life Sciences, Tewksbury, MA, USA), and the medium was replaced after cell adherence (approximately 3-4h). KCs were maintained in RPMI 1640 medium, and hepatocytes were maintained in DMEM/F12 (both from Gibco, Carlsbad, CA, USA) supplemented with 10% FBS at 37°C under an atmosphere with 5% CO_2_. The primary cells were then treated with 10 mM APAP (dissolved in phosphate buffer solution (PBS) at 37°C) for 12 h. All cell treatments were performed in a blinded and random manner.

### Western Blotting

All western blotting procedures were performed as previously described (30) with the following primary antibodies: anti-PRX3, anti-NLRP3 (Abclonal Biotechnology, Wuhan, China), anti-gasdermin D (GSDMD), anti-caspase-1, anti-cleaved caspase-1, anti-IL-1β (all from Cell Signaling Technology, Boston, MA, USA), anti-IL-18, anti-PRX5 (Abcam, Cambridge, UK), PRX6 (Zen bio., Chengdu, China) and anti-β-actin (Bimake, Houston, TX, USA).

### RNA Isolation and qRT-PCR

Total RNA was extracted using TRIzol reagent (Invitrogen, Carlsbad, CA, USA). Then, cDNA synthesis and qRT-PCR were performed with a PrimeScriptTM RT reagent kit and SYBR Premix Ex TaqTM II (TaKaRa, Japan), respectively. Specific primers used for qRT-PCR were as follows: mmu-PRX3-FO, (5’-TGCTGGCATTGCACTCAGA-3’), mmu-PRX3-RE (5’-ACTTCTCCATGGGTCTCCACAA-3’), mmu-β-actin-FO (5’-ACTGCCGCATCCTCTTCCT-3’), and mmu-β-actin-RE (5’-TCAACGTCACACTTCATGATGGA-3’).

### Cell Transfection

Primary cells were isolated from mice liver and seeded into rat tail collagen I-coated culture plates (6-well) at a density of 10^6^ cells per well. The small interfering RNA (siRNA; 50 pM) or negative control (GenePharma, Suzhou, China) was respectively pre-mixed with Lipofectamine™ 3000 Reagent (3.75 μl per well; Invitrogen, Carlsbad, CA, USA) according to the manufacturer’s instructions. After incubating for 15 min at room temperature, the mixtures were added to adherent primary liver cells (approximately 50%) for 36h in the original medium. The PRX3-specific siRNA sequences were sense 5’-CCAAGGAAAGUCAGCCUUUTT-3’ and antisense 5’-AAAGGCUGACUUUCCUUGGTT-3’. The NLRP3-specific siRNA sequences were sense 5’-CCAACUGGUCAAGGAGCAUTT-3’ and antisense 5’-AUGCUCCUUGACCAGUUGGTT-3’. The PRX5-specific siRNA sequences were sense 5’-GGCCGGAAAGAAGCAGGUUTT-3’ and antisense 5’-AACCUGCUUCUUUCCGGCCTT-3’. The PRX6-specific siRNA sequences were sense 5’-GGACGCUAACAACAUGCCUTT-3’ and antisense 5’-AGGCAUGUUGUUAGCGUCCTT-3’. The expression plasmid (1 μg/ml) or negative control plasmid (GenePharma) was respectively pre-mixed with Lipofectamine™ 3000 Reagent (3.75 μl per well) and P3000™ Reagent (5 μl per well) for 15 min following the manufacturer’s instructions. Then, plasmid DNA-lipid complexes were transfected into adherent primary hepatocytes (approximately 50%) for 48 h in the original medium. After transfection, the cells were incubated with APAP (10 mM) for 12 h for different experiments.

### Mitochondrial ROS Level Determination

Mito-TEMPO is a mitochondria-targeted antioxidant, a specific scavenging agent for mitochondrial superoxides. Hepatocytes were stimulated with 200 μM Mito-TEMPO (dissolved in DMSO) for 1 h, followed by treatment with 10 mM APAP for 12 h.

Mitochondrial ROS levels were detected by visualization with MitoSOX Red (Invitrogen, Carlsbad, CA, USA). Briefly, primary mouse hepatocytes were fixed on a six-well plate and incubated with 5 μM MitoSOX Red for 10 min at 37°C in the dark. The nuclei were recognized by Hoechst 33342 staining (Beyotime, Shanghai, China) in the dark for 5-10 min. Immunofluorescence images were obtained under an 80iNikon confocal microscope.

### Double Immunofluorescence Microscopy

After fixation in 4% paraformaldehyde, primary hepatocytes were incubated with primary antibody at 4°C overnight, followed by an incubation with secondary antibody (Proteintech, Wuhan, China) at 37°C for 1 h. Subsequently, Hoechst 33342 was used for nuclear staining. Immunofluorescence images were captured with an 80i Nikon confocal microscope.

### H_2_O_2_ Analysis

H_2_O_2_ levels were examined using a hydrogen peroxide assay kit (Solarbio, Beijing, China) according to the manufacturer’s recommended protocol.

### Statistical Analysis

All data are presented as the means ± standard deviation (SD). Student’s unpaired t-test (two-group comparisons) and one-way ANOVA (multigroup comparisons) were carried out using GraphPad Prism version 7.0 (GraphPad Prism Software, CA, USA). A p-value of < 0.05 was considered to indicate a significant difference.

## Results

### Pyroptosis Occurs in Mice With APAP-Induced Liver Injury

**Figure 1:**
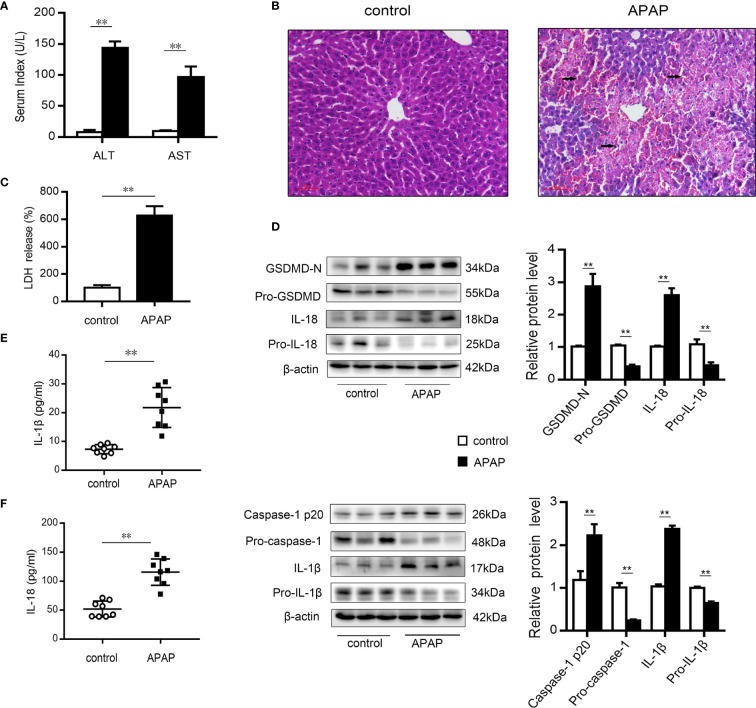
Pyroptosis occurs in mice with APAP-induced liver injury. Liver and serum samples were obtained from mice treated with APAP (300 mg/kg) for 12 h. **(A)** Serum levels of ALT and AST were assessed, n=8. **(B)** Liver sections were stained with H&E (50 μm), and the damaged area are indicated with arrows. **(C)** Serum levels of LDH were assessed, n=8. **(D)** Pro-GSDMD/GSDMD-N, pro-caspase-1/caspase-1 p20, pro-IL-1β/IL-1β, and pro-IL-18/IL-18 protein levels in mice liver were determined, n=3. **(E)** Serum levels of IL-1β. **(F)** Serum levels of IL-18, n=8. **P<0.01.

After excess APAP administration, the serum levels of ALT and AST in mice were clearly elevated (**Figure 1A**). Histological H&E staining revealed that central lobular necrosis, central venous disappearance, and disordered cell arrangement were severe and widespread in the liver of APAP-treated mice (**Figure 1B**). Notably, in the APAP group, the serum LDH content was increased by 6.5-fold compared to that observed in the wild-type mice group (**Figure 1C**), suggesting that a breach in cell membrane integrity and the efflux of cellular contents occurred after APAP stimulation (35). To assess the potential role of pyroptosis in the pathogenesis of APAP-induced hepatotoxicity, we further examined the protein levels of GSDMD, caspase-1, IL-1β, and IL-18, the key proteins in pyroptosis regulation (17, 35, 36). As shown in **Figure 1D**, increased levels of the N-terminal fragment of GSDMD (GSDMD-N) were found in liver tissue of APAP model mice. Similarly, the expression of the precursor of caspase-1 was downregulated, while that of the active fragment of caspase-1 (p20) was significantly upregulated, which resulted in the cleavage and activation of GSDMD. During this period, protein levels and serum levels of the proinflammatory cytokines IL-1β and IL-18 were increased after APAP intervention (**Figures 1E, F**). These results suggest that pyroptosis is related to APAP-induced liver injury.

### Primary KCs and Hepatocytes Undergo Pyroptosis Upon APAP Treatment

**Figure 2:**
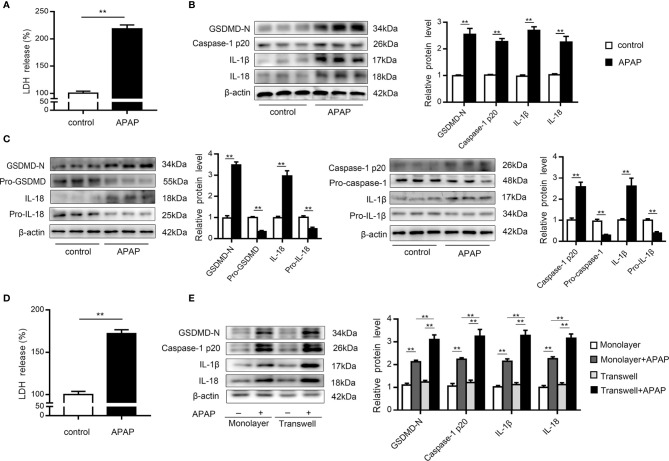
Primary KCs and hepatocytes undergo pyroptosis upon APAP treatment. Primary cells were plated on rat tail collagen-coated plates, and APAP (10 mM/well) was administered after stable adherence. **(A)** Kupffer cellular supernatant levels of LDH were assessed, n=6. **(B)** GSDMD-N, caspase-1 p20, IL-1β, and IL-18 protein levels in primary KCs were determined, n=3. **(C)** Pro-GSDMD/GSDMD-N, pro-caspase-1/caspase-1 p20, pro-IL-1β/IL-1β, and pro-IL-18/IL-18 protein levels in primary hepatocytes were determined, n=3. **(D)** Hepatocellular supernatant levels of LDH were assessed, n=6. **(E)** Primary hepatocytes were obtained from untreated C57BL/6 mice and reseeded in a monolayer or the lower chamber of a transwell system. For coculture, primary KCs from untreated C57BL/6 mice were reseeded in the upper chamber of the transwell system. The levels of the indicated proteins (GSDMD-N, caspase-1 p20, IL-1β, IL-18) in the primary hepatocytes from the monolayer or transwell cultures were detected by western blotting, n=3. **P<0.01.

KCs are rapidly lost after APAP overdose, which is accompanied by inflammatory cell infiltration (9, 10). To investigate whether pyroptosis contributes to KCs death, we performed western blot analysis of primary KCs exposed to APAP. The results showed that LDH release increased in the supernatants of KCs treated with APAP (**Figure 2A**). Notably, cleaved caspase-1 (p20) expression was dramatically elevated, causing an increase in GSDMD-N levels. Additionally, mature IL-1β and IL-18 levels were upregulated by approximately 2.5- and 2-fold, respectively, in the APAP group compared to the control group (**Figure 2B**). These results suggested that the massive loss of KCs after APAP intervention may have been partly caused by pyroptosis. Furthermore, massive death of hepatocytes is a key event after APAP overdose (8). To verify the association between pyroptosis and hepatocyte death, we assessed LDH and pyroptosis-associated protein levels in primary hepatocytes exposed to APAP. As shown in **Figure 2D**, increased LDH release was detected in APAP-treated hepatocellular supernatants. The precursor protein expression levels of GSDMD, caspase-1, IL-1β and IL-18 were decreased in the APAP group compared to the untreated group, while APAP administration led to substantial upregulation of the levels of the active fragments of these proteins. The dynamic change from the original to the activated forms of these key proteins indicate the occurrence of pyroptosis in primary hepatocytes incubated with APAP (**Figure 2C**).

It is important to note that increasing attention has been paid to the intercellular communication between KCs and hepatocytes (11, 37, 38). After APAP stimulation, we observed that caspase-1 p20 and GSDMD-N levels were upregulated in hepatocytes cocultured with KCs compared to monolayer hepatocytes, with increases in the levels of the inflammatory factors IL-1β and IL-18 also detected (**Figure 2E**). These data suggest that after excessive APAP intake, hepatocytes exposed to KCs underwent more pyroptosis.

### PRX3 Silencing Accelerates Pyroptosis in APAP-Induced Hepatotoxicity in Mice

Mitochondrial oxidative stress and intracellular inflammation are generally associated with APAP toxicity (2, 3, 25, 28, 39). To assess the protective effect of the PRX family members on APAP hepatotoxicity, specific siRNAs for three mitochondria-associated PRXs (PRX3, PRX5 and PRX6) (25, 40) were transfected into hepatocytes prior to APAP-induced cytotoxicity (**Figures S1A–C**). As shown in **Figures S1D, E**, we observed that PRX3 silencing resulted in more mtROS and increased inflammatory marker levels (IL-1β and IL-18) compared to knockdown of PRX5 and PRX6 after APAP intervention. These results indicate that PRX3 is a protective target among mitochondria-associated PRXs to prevent APAP-induced hepatotoxicity.

**Figure 3:**
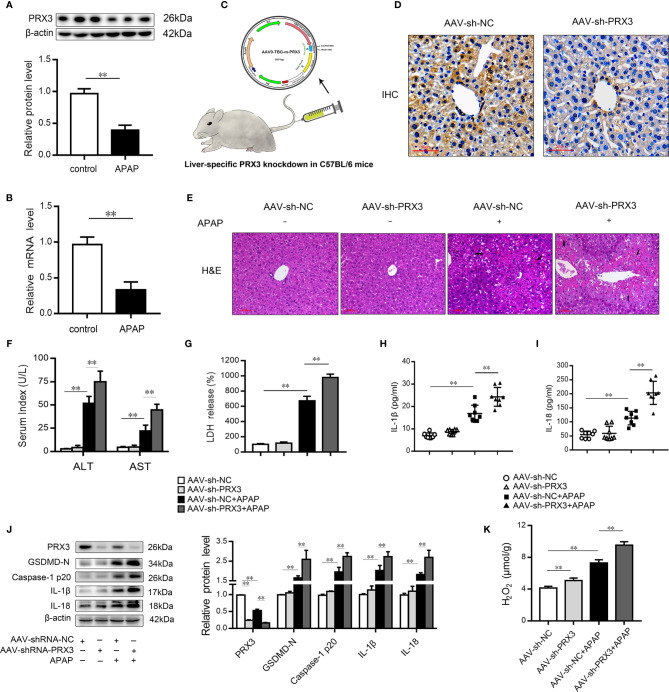
PRX3 silencing accelerates pyroptosis in APAP-induced hepatotoxicity in mice. **(A)** PRX3 protein levels in mice liver were determined, n=3. **(B)** Liver PRX3 mRNA expression, n=6. **(C)** Schematic representation of AAV9-TBG-shRNA injection through tail vein in mice. **(D)** Liver sections were analyzed by immunohistochemical staining for PRX3 (50μm). **(E)** Liver sections were stained with H&E (50μm). The damaged areas are indicated with arrows. **(F)** Serum levels of ALT and AST were assessed, n=8. **(G)** Serum levels of LDH were assessed, n=8. **(H, I)** Serum levels of IL-1β and IL-18, n=8. **(J)** PRX3, caspase-1 p20 and GSDMD-Nin mice liver were determined, n=3. **(K)** H_2_O_2_ level, n = 8.**P<0.01.

We subsequently evaluated the expression of PRX3 in the livers of mice following APAP administration. As shown in **Figures 3A, B**, PRX3 protein and mRNA levels were significantly decreased after APAP intoxication compared to the control group. To systematically assess the function of PRX3 in APAP-induced liver injury, we generated PRX3 liver-specific knockdown mice by AAV9-shRNA-PRX3 treatment (**Figure 3C**). IHC and western blotting results indicated that PRX3 expression was notably inhibited by AAV9-shRNA-PRX3 (**Figures 3D–J**). According to the H&E staining, treatment with PRX3 silencing promoted characteristic centrilobular necrosis after APAP overdose (**Figure 3E**). Consistent with these results, the serum levels of ALT, AST and LDH release revealed that PRX3 knockdown aggravated APAP-induced liver injury (**Figures 3F, G**). We further assessed the impact of PRX3 in APAP-induced liver pyroptosis. Liver specific PRX3 silencing markedly increased serum levels of IL-1β and IL-18 as well as liver H_2_O_2_ levels in mice treated with APAP, indicating fulminant oxidative stress and inflammation (**Figures 3H, I, K**). Additionally, as shown in **Figure 3J**, we observed that AAV9-shRNA-induced PRX3 knockdown significantly upregulated GSDMD, caspase1 p20, IL-1β and IL-18 levels, the specific markers of pyroptosis. Taken together, these results demonstrate that PRX3 is associated with pyroptosis in APAP-induced hepatotoxicity.

### PRX3 Regulates NLRP3-Mediated Pyroptosis in APAP-Induced Hepatotoxicity *In Vitro*

**Figure 4:**
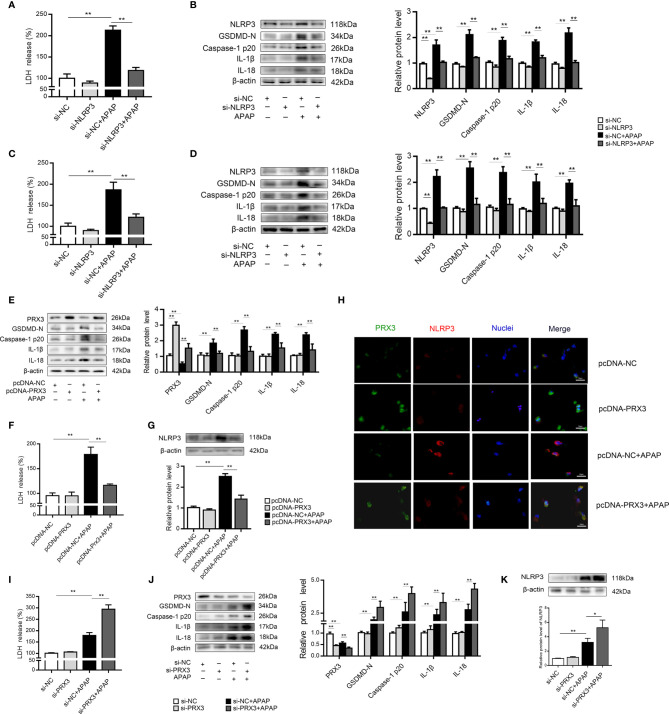
PRX3 regulates NLRP3-mediated pyroptosis in APAP-induced hepatotoxicity *in vitro.* Primary KCs and hepatocytes were transfected with NLRP3-specific siRNA and then exposed to APAP (10 mM). **(A)** Kupffer cellular supernatant levels of LDH were assessed, n=6. **(B)** NLRP3, GSDMD-N, caspase-1 p20, IL-1β, and IL-18 protein levels in primary KCs, n=3. **(C)** Hepatocellular supernatant levels of LDH were assessed, n=6. **(D)** NLRP3, GSDMD-N, caspase-1 p20, IL-1β, and IL-18 protein levels in primary hepatocytes, respectively, n=3. **(E)** Primary hepatocytes were transfected with pcDNA-PRX3 and then exposed to APAP (10 mM). PRX3, GSDMD-N, caspase-1 p20, IL-1β, and IL-18 protein levels in primary hepatocytes were determined, n=3. **(F)** Hepatocellular supernatant levels of LDH were assessed, n=6. **(G)** NLRP3 protein levels in primary hepatocytes, n=3. **(H)** Double immunofluorescence staining for PRX3 and NLRP3 in primary hepatocytes, 50 μm. **(I)** Hepatocellular supernatant levels of LDH were assessed, n=6. **(J)** Primary hepatocytes were transfected with si-PRX3 and then exposed to APAP (10 mM). PRX3, GSDMD-N, caspase-1 p20, IL-1β and IL-18 protein levels in primary hepatocytes were determined, n=3. **(K)** NLRP3 protein levels in primary hepatocytes, n=3. **P<0.01, *P<0.05.

We further investigated the underlying mechanisms of APAP-induced liver pyroptosis. NLRP3 inflammasome activation is regarded as a major event in the initiation of pyroptosis (16, 23). As shown in **Figure S2A**, NLRP3 expression in mice was markedly increased after APAP treatment. Consistently, we observed the same trend *in vitro* (**Figures S2B, C**). Interestingly, NLRP3-specific siRNAs rescued APAP-induced cell death determined by LDH release in supernatant of KCs and hepatocytes (**Figures 4A, C**). Furthermore, we found that si-NLRP3 resulted in a notable recovery of cleaved GSDMD, caspase-1, IL-β and IL-18 expression induced by APAP treatment (**Figures 4B, D**). Thus, NLRP3 contributes to the pathogenesis of APAP-induced liver pyroptosis.

In primary KCs and hepatocytes exposed to APAP, PRX3 protein levels showed the same downward tendency (**Figures S2D, E**). To further assess whether PRX3 plays a role in NLRP3-dependent pyroptosis *in vitro*, hepatocytes were transfected with PRX3-specific pcDNA or siRNA prior to APAP intoxication. Double immunofluorescence staining confirmed that PRX3 (green) and NLRP3 (red) were both located in the mitochondria and highly colocalized (**Figure 4H**). Notably, overexpression of PRX3 reduced the activation-responsive protein expression of pyroptotic genes, impaired NLRP3 inflammasome activation and inhibited LDH release (**Figures 4E–G**), while knockdown of PRX3 significantly enhanced NLRP3 inflammasome activation and stimulated pyroptosis (**Figures 4I–K**). Thus, our data indicate that PRX3 regulates APAP-associated pyroptosis through the NLRP3 inflammasome.

### PRX3 Regulates the NLRP3 Inflammasome Through Mitochondrial ROS After APAP Overdose

**Figure 5:**
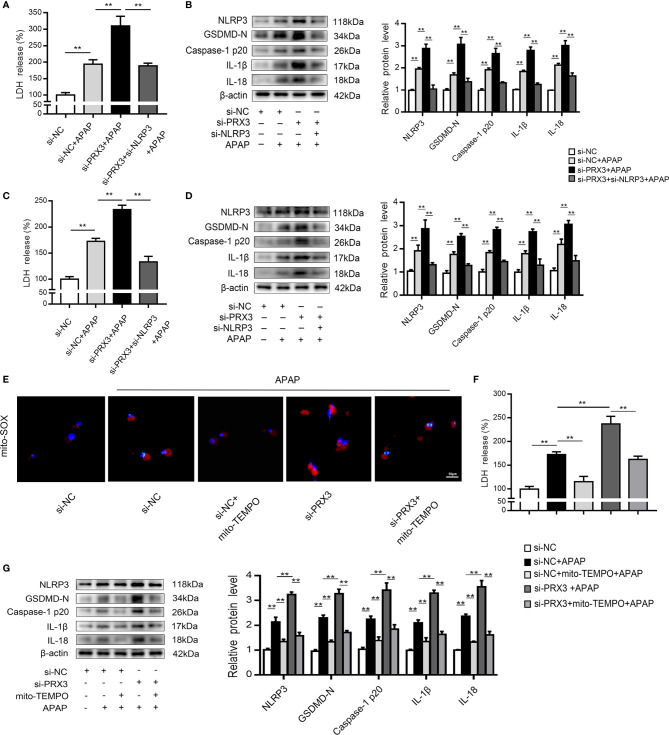
PRX3 regulates the NLRP3 inflammasome through mitochondrial ROS after APAP overdose. Primary KCs and hepatocytes were cotransfected with PRX3- and NLRP3-specific siRNAs following APAP challenge. **(A)** Kupffer cellular supernatant levels of LDH were assessed, n=6. **(B)** NLRP3, GSDMD-N, caspase-1 p20, IL-1β and IL-18 protein levels in primary KCs n=3. **P<0.01. **(C)** Hepatocellular supernatant levels of LDH were assessed, n=6. **(D)** NLRP3, GSDMD-N, caspase-1 p20, IL-1β and IL-18 protein levels in primary hepatocytes, n=3. **P<0.01. PRX3 suppression was applied in primary hepatocytes via PRX3-specific siRNA transfection and subsequent Mito-TEMPO (0.2 mM) treatment before APAP intervention. **(E)** Representative fluorescence images of MitoSOX, 50 μm. **(F)** hepatocellular supernatant levels of LDH were assessed, n=6. **(G)** NLRP3, GSDMD-N, caspase-1 p20, IL-1β, and IL-18 protein levels in primary hepatocytes, n=3. **P<0.01.

To further investigate the regulatory mechanism between PRX3 and NLRP3, KCs and hepatocytes were co-transfected with PRX3- and NLRP3-specific siRNAs prior to APAP overdose. As shown in **Figures 5A, B**, PRX3 knockdown aggravated the triggering effect of NLRP3 on pyroptosis (including the increased serum level of LDH and protein levels of GSDMD-N, caspase-1 p20, IL-1β and IL-18) in KCs, which was vastly attenuated by NLRP3-specific siRNA. Additionally, we observed the same results in hepatocytes (**Figures 5C, D**). Taken together, our results suggest that PRX3 is a crucial upstream mediator of NLRP3 in the regulation of pyroptosis.

Mitochondrial ROS play a vital role in NLRP3 inflammasome activation (22), and ROS can be inhibited by PRX3 through its antioxidant function (28). To further investigate the relationship between PRX3 and NLRP3, we transfected hepatocytes with PRX3-specific siRNA in the absence/presence of Mito-TEMPO (a specific mtROS scavenger). Under APAP stimulation, PRX3 knockdown resulted in abundant mtROS production in hepatocytes, which was blocked by Mito-TEMPO, as detected using Mito-SOX (**Figure 5E**). Furthermore, the decreased mtROS levels resulting from Mito-TEMPO treatment substantially attenuated the activation of NLRP3, caspase-1 and GSDMD, as well as the secretion of IL-1β, IL-18 and LDH (**Figures 5F, G**). Considering all of the above data, these results suggest that the effect of PRX3 on NLRP3 activation in APAP-induced liver pyroptosis is potentially mediated by mtROS.

## Discussion

**Figure 6:**
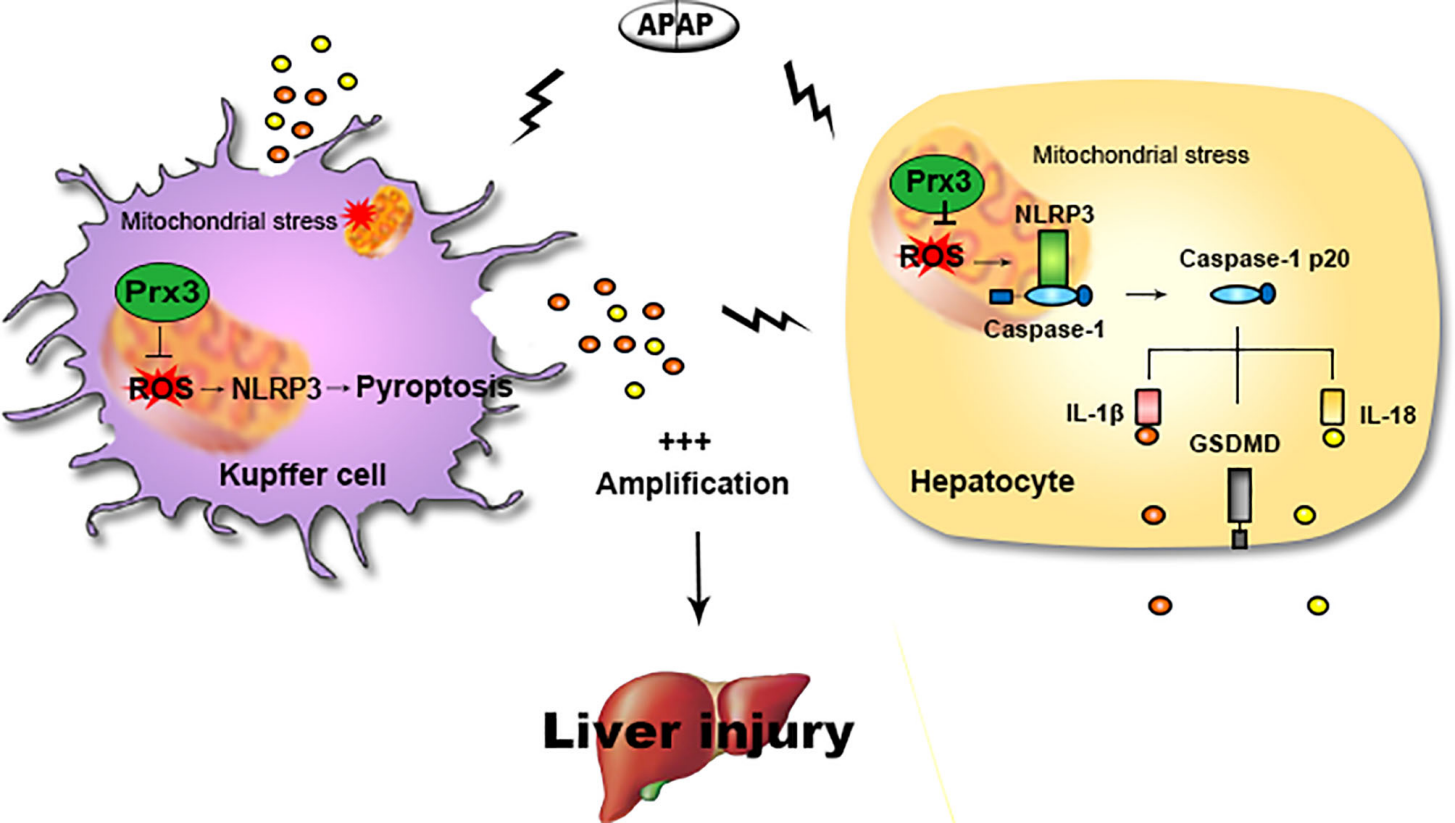
Illustration of the mechanism by which PRX3 alleviates APAP-induced pyroptosis. Excessive APAP promotes NLRP3-mediated pyroptosis in mice. The crosstalk between KCs and hepatocytes amplifies the pyroptotic signal. PRX3 overexpression alleviates the above process by disrupting NLRP3 inflammasome activation. Moreover, PRX3 regulates NLRP3 inflammasome activation by targeting mitochondrial ROS.

The main findings of this study are as follows: (1) NLRP3-mediated pyroptosis contributes to APAP-mediated liver injury, while cellular crosstalk between KCs and hepatocytes amplifies the signal for pyroptosis; (2) PRX3 silencing aggravates APAP-induced liver pyroptosis and injury *in vivo* and *in vitro*; (3) PRX3 confers protection against APAP-induced pyroptosis by inhibiting NLRP3 inflammasome activation; and (4) PRX3 regulates the NLRP3 inflammasome by targeting mitochondrial ROS (**Figure 6**). These findings suggest that PRX3 may be a new therapeutic target in pyroptosis in the context of APAP-induced acute liver injury.

APAP-induced hepatotoxicity is a leading cause of hepatic failure involving multiple intracellular events, including mitochondrial damage, oxidative stress, and inflammation (41). Especially, secondary multiorgan failure, which is caused by APAP toxicity, is often a result of the initial massive proinflammatory response and generates a systemic inflammatory response syndrome (42). As an inherently inflammatory process, pyroptosis plays vital roles in cellular lysis and proinflammatory cytokine release when hosts defend against infections (43). However, pyroptosis has not been clearly described in APAP-induced liver injury (14). The viewpoint of pyroptosis in APAP poisoning was controversial, possibly because Imaeda (44) and Williams (45) held opposite views on the role of NLRP3 and caspase-1 in APAP-induced liver injury. Thus, additional evidence is needed to investigate whether pyroptosis is involved in APAP hepatotoxicity. In the present study, we observed that upregulation of LDH release occurred concurrently with liver damage, which suggests that cellular damage occurs after APAP stimulation. More importantly, the levels of the active fragments of pyroptosis-related proteins (GSDMD-N, caspase-1 p20, IL-1β and IL-18) greatly increased upon APAP exposure *in vivo* and *in vitro*. Pyroptosis is induced by the plasma membrane insertion of large pre-assembled non-selective GSDMD-N pores that allowed IL-1β and IL-18 to be released into the blood, giving rise to rapid cellular swelling and lysis (46). Indeed, we observed that APAP overdose gave rise to a massive release of IL-1β and IL-18 into the serum of mice by ELISA. In addition, when exposed to KCs, hepatocytes underwent much more pyroptosis after APAP intervention, which is consistent with the view of Li Y Z et al. (47). Therefore, pyroptosis is closely associated with APAP-induced liver injury.

PRX3 is primarily located in the mitochondria and plays crucial roles in the reduction and clearance of H_2_O_2_ and peroxynitrite (26). Recent studies have demonstrated that PRX3 knockdown increases mitochondrial superoxide levels (48) and induces the activation of RAW264.7 macrophages (28). Furthermore, relatively high levels of ROS and TNF-α accumulation have been detected in PRX3-deficient peritoneal macrophages (49), indicating that PRX3 is associated with inflammation. Notably, we observed that pyroptosis was involved in APAP-induced hepatotoxicity, and was accompanied by increasing levels of inflammatory factor. In light of these studies, we hypothesized that the antioxidative capacity of PRX3 is associated with the regulation of pyroptosis. Interestingly, our results demonstrated that PRX3 levels decreased upon APAP challenge. Additionally, upon APAP intoxication, PRX3 silencing aggravated liver pyroptosis and injury compared to the control group *in vivo* and *in vitro*. In contrast, enhanced levels of pyroptotic markers stimulated by APAP overdose were reversed by PRX3 overexpression *in vitro*. Hence, PRX3 regulates APAP-induced liver pyroptosis.

We further investigated the mechanism by which PRX3 regulates APAP-induced pyroptosis by assessing NLRP3 inflammasome activation, which has been revealed by increasing evidence to be a key promoter of pyroptosis in several diseases (18–21). Our results confirmed that NLRP3 knockdown led to the suppression of pyroptosis during APAP-induced hepatotoxicity *in vitro*. Mitochondria have been reported to act as docking sites for NLRP3 inflammasome assembly (23) and our subsequent observations revealed that PRX3 colocalized with NLRP3 in the mitochondria. Moreover, PRX3 overexpression was shown to impair NLRP3 inflammasome activation upon exposure to APAP, while PRX3 knockdown enhanced NLRP3 inflammasome activation. Moreover, our data showed that pyroptosis caused by PRX3 deficiency was antagonized by NLRP3-specific siRNA. Collectively, these results confirm that PRX3 may be responsible for modulating the NLRP3 inflammasome and, ultimately, for the initiation of pyroptosis. Excessive mitochondrial ROS promotes the initiation of NLRP3 inflammasome activation (50). After APAP overdose, we observed that the treatment of primary hepatocytes with Mito-TEMPO blocked the PRX3 knockdown-induced release of mitochondrial ROS and the high NLRP3 expression, which simultaneously resulted in the decreased expression of pyroptosis-associated proteins. These results indicate that the anti-pyroptotic effect of PRX3 in the context of APAP-induced hepatotoxicity is associated with suppression of NLRP3 inflammasome activation and is mediated by inhibiting mitochondrial oxidative stress.

Our findings demonstrate that oxidative stress and pyroptosis are not independent but rather interact to facilitate the complexity of APAP-induced liver injury, which is partly consistent with the view of Diao (51). PRX3 plays a major role in mitochondrial redox signaling (25) and is a crucial factor in various diseases, such as colon cancer, chronic hyperglycemia and hepatocellular carcinoma (52–54). In the present study, for the first time, we demonstrated that PRX3 overexpression mitigates APAP-induced liver pyroptosis, although this alleviation may occur through other pathological processes, which needs to be further investigated. At present, N-acetylcysteine (NAC) is the only currently approved antidote for APAP overdose. Unfortunately, fairly high doses and prolonged treatment times are required due to the poor bioavailability of NAC and the associated anaphylactoid reactions (55, 56). Therefore, the development of therapeutic strategies to regulate PRX3 function may represent a more effective alternative to combat APAP-induced hepatotoxicity. The considerable potential of human Prx3 (hPrx3) as a tecton for use in protein nanotechnology has been previously demonstrated. hPrx3 forms toroidal oligomers characteristic of the PRX family and shows an amazing array of supramolecular assemblies (57), indicating the potential use of PRX3 in the development of specific and novel drugs to treat liver injury.

In summary, the results of our present study demonstrated for the first time, that PRX3 inhibits APAP-induced pyroptosis by inhibiting NLRP3 activation, which depends on the increase in mitochondrial ROS levels. These findings identify the crucial regulatory role of PRX3 in APAP-induced liver damage and provides novel insights into the pathological mechanisms of APAP-induced liver injury.

## Data Availability Statement

The original contributions presented in the study are included in the article/**Supplementary Material**. Further inquiries can be directed to the corresponding authors.

## Ethics Statement

The animal study was reviewed and approved by The Institutional Ethics Committee of Dalian Medical University.

## Author Contributions

JY, XT, and YW designed the study. YW, YZ, ZW, DL, and RS performed the experiments. ML, BZ, and RL analyzed the data. YW, JZ, and SN contributed experimental materials. JY, XT, and YW prepared the manuscript. JY, YZ, ML, and SN provided financial support. All authors contributed to the article and approved the submitted version.

## Funding

This study was funded by the National Natural Science Foundation of China (Nos. 81973381, 81773799, 82004044 and 81903900), Liaoning Provincial Natural Science Foundation of China (Nos. 20180551172), and Dalian Medical Science Research (Nos. 1712044).

## Conflict of Interest

The authors declare that the research was conducted in the absence of any commercial or financial relationships that could be construed as a potential conflict of interest.

## Supplementary Material

Supplementary Figure 1 Primary hepatocytes were transfected with specific siRNAs of PRX3, PRX5 and PRX6 prior to APAP-induced cytotoxicity. **(A–C)** PRX3, PRX5 and PRX6 knockdown levels in primary hepatocytes, n=3. **P<0.01 **(D)** IL-1β and IL-18 protein levels in primary hepatocytes, n=3. **P <0.01 vs. si-NC group. ns, ^#^P <0.05 and ^##^P <0.01 vs. si-NC + APAP group. **(E)** Representative fluorescence images of MitoSOX (50 μm).

Supplementary Figure 2
**(A)** NLRP3 protein levels in mice liver, n=3. **(B)** NLRP3 protein levels in primary KCs, n=3. **(C)** NLRP3 protein levels in primary hepatocytes, n=3. **(D)** PRX3 protein levels in primary KCs were determined, n=3. **(E)** PRX3 protein levels in primary hepatocytes were determined, n=3. **P<0.01.

## Abbreviations

APAP, acetaminophen; DILI, drug-induced liver injury; PRX3, peroxiredoxin 3; ROS, reactive oxygen species; mtROS, mitochondrial ROS; KCs, kupffer cells; ALF, acute liver failure; NAPQI, n-acetyl-p-benzoquinone imine; PCD, programmed cell death; I/R, ischemia/reperfusion; ALT, aminotransferase; AST, aspartic aminotransferase; LDH, lactate dehydrogenase; OD, optical density; H&E, hematoxylin-eosin; IHC, immunohistochemistry; siRNA, small interfering RNA; GSDMD, gasdermin D; GSDMD-N, N-terminal fragment of GSDMD; NAC, n-acetylcysteine; NLRP3, nod-like receptor protein 3; IL-1β, interleukin-1 beta; IL-18, interleukin-18.
