# Supplementary figures and images for: Peroxiredoxin 3 Inhibits Acetaminophen-Induced Liver Pyroptosis Through the Regulation of Mitochondrial ROS
Source: Front Immunol. 2021 May 13;12:652782. doi: 10.3389/fimmu.2021.652782 (PMC8155593; doi:10.3389/fimmu.2021.652782)

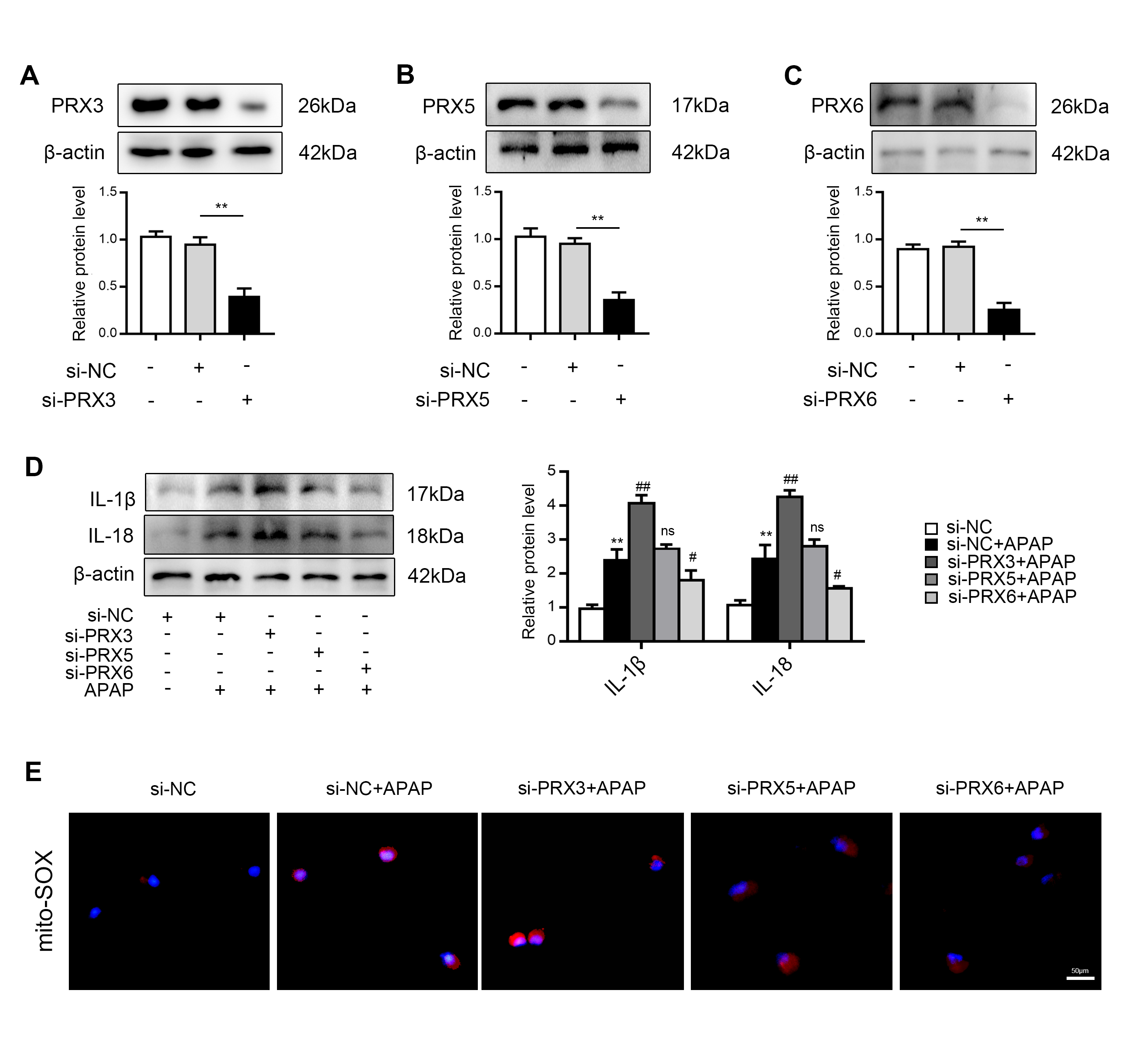

Supplement: Supplementary Figure 1 — Primary hepatocytes were transfected with specific siRNAs of PRX3, PRX5 and PRX6 prior to APAP-induced cytotoxicity. (A–C) PRX3, PRX5 and PRX6 knockdown levels in primary hepatocytes, n=3. **P<0.01 (D) IL-1β and IL-18 protein levels in primary hepatocytes, n=3. **P <0.01 vs. si-NC group. ns, #P <0.05 and ##P <0.01 vs. si-NC + APAP group. (E) Representative fluorescence images of MitoSOX (50 μm). [file Image_1.tif]

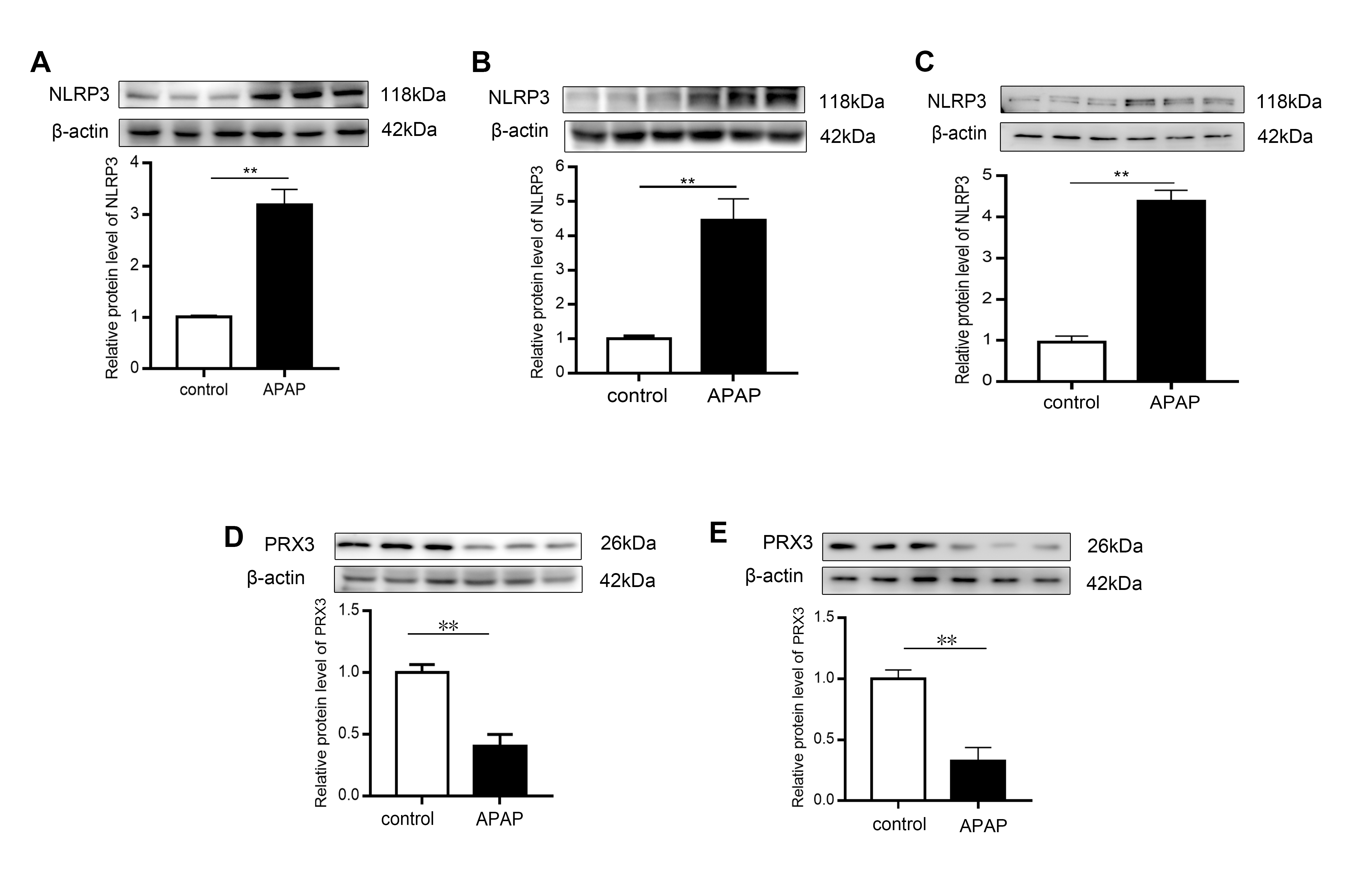

Supplement: Supplementary Figure 2 — (A) NLRP3 protein levels in mice liver, n=3. (B) NLRP3 protein levels in primary KCs, n=3. (C) NLRP3 protein levels in primary hepatocytes, n=3. (D) PRX3 protein levels in primary KCs were determined, n=3. (E) PRX3 protein levels in primary hepatocytes were determined, n=3. **P<0.01. [file Image_2.tif]
